# Viral Strategies and Cellular Countermeasures That Regulate mRNA Access to the Translation Apparatus

**DOI:** 10.3390/v17060766

**Published:** 2025-05-28

**Authors:** Christopher U. T. Hellen

**Affiliations:** Department of Cell Biology, SUNY Downstate Health Sciences University, Brooklyn, NY 11203, USA; christopher.hellen@downstate.edu

**Keywords:** translational control, mRNA translation, IRES, interferon-stimulated gene, Shiftless, ITAF, picornavirus, calicivirus, coronavirus, poxvirus

## Abstract

The papers introduced in the Commentary present new insights and review aspects of current knowledge concerning the competition between viruses and their hosts for the cellular translation apparatus. Viruses depend on this apparatus and utilize diverse mechanisms to usurp it for the translation of viral mRNAs and to suppress synthesis of cellular proteins. Virus-induced modification of translation factors, selective abrogation of mRNA binding to ribosomes and degradation of cellular mRNAs all impair elements of the innate immune response, thereby undermining host defenses against infection. Various cellular mechanisms prevent translation of viral mRNAs, by modifying components of the translation apparatus to effect a generalized shut-off of translation or by binding of host proteins to viral mRNAs to induce their degradation or to prevent their engagement with the translation apparatus. Viruses have in turn evolved countermeasures to evade these defenses, for example by encoding proteins that impair the activity of host factors or via alterations in the sequence and structure of viral mRNAs. Such changes enable viral mRNAs to avoid recognition by host factors or to support translation initiation by specialized mechanisms that involve only a subset of the factors that are required by cellular mRNAs.

Viruses depend on the translation apparatus of host cells for the synthesis of viral proteins. Cells employ various countermeasures to restrict the translation of viral mRNAs, and viruses have responded to these cellular defenses by evolving mechanisms to evade them. The outcome of viral infection is determined by these competing processes. In this Special Issue, leading experts present and review data regarding new developments in the understanding of mechanisms by which viruses exploit the cellular translation apparatus and counter or evade cellular mechanisms that restrict viruses.

## 1. Mechanism of mRNA Translation in Eukaryotes

Translation consists of four stages: initiation, elongation, termination, and ribosome recycling. During the canonical initiation process [1], an mRNA with a 5′-terminal m^7^G cap and a 3′-terminal poly(A) tail is selected by eukaryotic initiation factor (eIF) 4F, a heterotrimer that consists of eIF4E (the cap-binding subunit), eIF4A (an RNA helicase), and eIF4G (which binds to eIF4E, eIF4A, eIF3, and the poly(A)-binding protein (PABP)). Initiator tRNA (Met-tRNA_i_^Met^) forms a ternary complex with eIF2•GTP, and it binds with eIF1, eIF1A, and eIF3 to a ribosomal 40S subunit to form a 43S preinitiation complex. This complex is recruited to the capped 5′-end of the mRNA by eIF4F and scans downstream, and when it encounters an initiation codon, eIF2-bound GTP is hydrolyzed and initiator tRNA is released into the ribosomal peptidyl (P) site to form a 48S complex. eIF5B promotes the joining of a 60S ribosomal subunit to this complex, yielding an 80S ribosome that can begin elongation. eIF2B recycles eIF2•GDP to eIF2•GTP for the next round of initiation. Elongation involves cycles of delivery of cognate aminoacyl-tRNA to the ribosomal aminoacyl (A) site by eukaryotic elongation factor (eEF) 1A, peptide bond formation, and translocation promoted by eEF2 of the base-paired mRNA and peptidyl-tRNA from A to P sites. The entry of a stop codon into the A site signals the termination of translation, which involves the binding of eRF1/eRF3•GTP to the A site, the recognition of the stop codon by eRF1, GTP hydrolysis, the release of eRF3•GDP, the cleavage of peptidyl-tRNA, and the release of the completed polypeptide [2]. The resulting post-termination complex, still bound to eRF1, is disassembled by ABCE1, freeing the 60S subunit. mRNA and deacylated tRNA are released from the 40S subunit by redundant pathways involving eIF1, eIF1A, and eIF3 or specific recycling factors (MCTS1/DENR or eIF2D). The 40S and 60S subunits can then participate in the next translation cycle.

## 2. Regulation of Translation by the Innate Immune Response

Host cells employ Toll-like, retinoic acid-inducible gene-I (RIG-I)-like, and other pattern-recognition receptors to detect viral nucleic acids and proteins, and to activate immune signaling pathways to induce the expression of interferon (IFN)-stimulated genes (ISGs). Specific elements of the innate immune response target different stages in the translation process to abrogate the translation of viral mRNAs, and in some instances, they also impair the translation of cellular mRNAs. Viruses have in turn developed diverse strategies to counteract or evade the activity of innate immune effectors.

Smart et al. [3] reviewed the activities of ISGs that target mRNAs, leading to the virus-specific abrogation of translation, or that target components of the translation apparatus, resulting in either the repression of viral translation or the activation of the translation of IFN-inducible antiviral proteins. Interferons induce the phosphorylation and consequent deactivation of the eIF4E-binding protein (4E-BP) by activating signaling pathways downstream of mammalian target of rapamycin (mTOR), leading to the release of eIF4E from 4E-BP, thereby promoting the translation of antiviral proteins. ISGs inhibit major stages in initiation, including the recruitment of mRNA and initiator tRNA to ribosomes. The interferon-induced proteins with tetratricopeptide repeats (IFITs) are abundantly expressed early in the antiviral response, and they have been reported to bind to eIF3 but also directly to non-self RNAs to inhibit their translation [4]. IFIT1 binds to the 5′-terminal region of mRNAs lacking methylation on the first cap-proximal nucleotide (‘cap0’), and its association with IFIT2-IFIT3 yields a high-affinity heterotrimeric complex that competitively inhibits the binding of eIF4F. Protein **K**inase R (PKR) phosphorylates eIF2α, causing it to sequester eIF2B, thereby limiting the recycling of eIF2•GDP to eIF2•GTP and globally repressing translation. PKR is one of several components of the integrated stress response that converges on the phosphorylation of eIF2α to repress translation. The synthesis of viral proteins in infected cells commonly overwhelms the endoplasmic reticulum (ER), activating the PKR-like ER kinase (PERK), which also phosphorylates eIF2α and downregulates viral translation. The phosphorylation of eIF2α is reversed by protein phosphatase 1, which is directed to p-eIF2α by the Constitutive R**e**pressor of eIF2α Phosphorylation (CReP) and by the stress-inducible Growth Arrest **a**nd DNA Damage-inducible 34 (GADD34). Zinc finger antiviral protein (ZAP) binds to viral mRNAs, with a preference for CpG-rich regions, and to eIF4A, impairing its interaction with eIF4G, preventing the formation of eIF4F, and impairing translation in a virus-specific manner. ZAP also promotes the degradation of some viral mRNAs and binds to coronavirus frameshift elements, impairing the −1 programmed ribosomal frameshifting that is required for the expression of replicase proteins. Shiftless (SHFL) impairs frameshifting on human immunodeficiency virus 1 (HIV-1) mRNA, and has other less-well-characterized inhibitory activities. Other ISGs directly impair the elongation process. Thus, Schlafen 11 is a tRNA endonuclease that cleaves leucine, serine, and other class II tRNAs, counteracting changes in the cellular tRNA repertoire induced by viral infection, thereby repressing the translation of specific viral mRNAs in a codon-usage-dependent manner. Other ISGs target viral mRNAs for degradation or modification, either directly or indirectly. These include ISG20, an RNAse, and adenosine deaminase acting on RNA 1 (ADAR1), which deamidates adenosines to inosines, leading to the miscoding of modified viral mRNAs. The 2′-5′ oligoadenylate synthase activates RNAse L, leading to the non-specific degradation of viral and cellular mRNAs.

## 3. Viral Mechanisms for Evasion of ISG-Mediated Repression of Translation

Different virus families have evolved strategies to evade aspects of ISG-mediated repression of translation by inhibiting the activity of individual ISGs or by exploiting alternative mechanisms for translation initiation. These strategies may involve the expression of viral proteins that inhibit or sequester ISGs or the acquisition of structural elements in viral mRNAs that enable them to exploit non-canonical initiation mechanisms. In either case, the lack of compatibility in different cell types may restrict viral growth, thus influencing tissue specificity and host range.

Alphaviruses such as Sindbis virus (SV) and Western equine encephalitis virus (WEEV) infect vertebrate hosts and arthropod (mosquito) vectors, causing moderate-to-high shut-off of host translation in mammalian cells but not in mosquitoes. Ventoso et al. [5] summarized the IFN-induced effectors that block host and viral translation in mammalian alphavirus-infected cells, principally PKR, ZAP, IFIT1, and IFIT3, and describe adaptive changes in alphavirus mRNAs that lead to the resistance to IFIT1 or ZAP. These changes include the presence of a stable 5′-terminal hairpin that impairs the binding of IFIT1, and the suppression of CpG content. Notably, such suppression is not evident in alphaviruses that infect only insects, which do not encode ZAP orthologs. Other structural features in alphaviruses confer resistance to the PKR-mediated phosphorylation of eIF2α by enabling an alternative, eIF2-independent mechanism of initiation.

PKR is an important factor in restricting viral infection [6], and its evasion is an important contributor to the tropism of poxviruses. They encode two proteins that suppress the activation of PKR: the vaccinia virus E3L protein and its orthologs bind dsRNA and prevent PKR dimerization and its consequent activation, whereas the K3L protein and its orthologs act as pseudo-substrates for PKR’s eIF2α-binding site. Megawati et al. [7] described the results of experiments performed to determine the ability of Yaba monkey tumor virus and Tanapox virus K3L orthologs to inhibit PKR from different primate species, and to restore the replication of a vaccinia virus strain lacking both E3L and K3L in different primate-derived cell lines. Their results showed that K3L inhibited PKR in primate cells in a species-dependent manner, and that K3L moieties from different viruses inhibit PKR in specific cell lines with varying efficiency. These findings provide insights into the influence of viral PKR antagonists as determinants of poxvirus host range.

Recent studies have established that coronaviruses influence the integrated stress response in different ways, ranging from the suppression of eIF2α phosphorylation by Middle East respiratory syndrome (MERS) coronavirus to its cell type-dependent activation by severe acute respiratory syndrome coronavirus 2 (SARS-CoV-2) [8]. Sodium arsenite is a strong inducer of PERK-mediated eIF2α phosphorylation, but Dolliver et al. [9] reported that this activity and the activation of PKR by polyinosinic-polycytidylic acid (poly(I:C), a dsRNA mimic) are both inhibited by human coronavirus OC43 (OC43) infection. Although GADD34 expression was induced by OC43, this was not responsible for the suppression of eIF2α phosphorylation in cells infected by this virus. Therefore, the mechanism by which OC43 influences this process remains to be determined.

Shiftless (SHFL) was identified as an inhibitor of −1 programmed ribosomal frameshifting (PRF) during the synthesis of the HIV-1 Gag-Pol precursor [10], but this activity is not essential for its role in inhibiting HIV infectivity [11]. The implication that SHFL may inhibit more than one facet of the translation process is consistent with the observation of its antiviral activity against viruses that are not dependent on frameshifting. Moreover, although SHFL expression is IFN-induced, it is expressed at a low level in a constitutive manner, suggesting that it may have cellular functions in addition to a role as an innate immune effector. Kelly and Dinman [12] reported the results of their investigation of the potential constitutive roles of SHFL that may have been co-opted to promote antiviral immunity. They reported that SHFL influences +1 PRF as well as −1 PRF, confirmed that it suppresses stop codon readthrough and that it promotes reporter gene expression, potentially by suppressing spontaneous frameshifting. Overexpression and silencing experiments indicated that SHFL influenced the stability of mRNA, and gene silencing experiments linked SHFL to the ribosome-associated quality control (RQC) process.

## 4. Structure of Viral mRNAs

The structures of viral mRNAs and the organization of their coding regions are influenced (a) by genome size (limited by the encapsidation process, and for many RNA viruses, by the potentially lethal accumulation of mutations in larger genomes due to the low fidelity of replication and the lack of proofreading), (b) by the need to accommodate *cis*-acting elements required for replication and for initiation of translation in a single molecule, and (c) by evolutionary changes in the sequence, structure, and post-transcriptional modifications of mRNAs that enables them to evade the innate immune restriction of viral translation. The constraint on size and characteristics of the canonical translation initiation process in eukaryotes favor genome compression for most RNA viruses.

Khan and Fox [13] discussed a selection of the structural and functional classes of RNA elements present in viral mRNAs. Several of these elements enable viral genomes to maximize the exploitation of the limited coding capacity of viral genomes, including alternative initiation codons and frameshifting signals for the translation of overlapping reading frames and internal ribosomal entry sites (IRESs), termination–reinitiation signals, and the synthesis of subgenomic mRNAs to enable the translation of 3′-terminal open reading frames. TURBS (Termination Upstream Ribosome Binding Site) elements, which occur in mRNAs of influenza B virus (*Orthomyxoviridae*), members of the genus *Hapavirus* of *Rhabdoviridae*, and members of several genera of *Caliciviridae* [14,15], are dedicated termination–reinitiation elements that subvert ribosome recycling by tethering post-termination 40S ribosomal subunits to mRNA to promote the reinitiation of translation on bicistronic viral mRNAs in the vicinity of the open reading frame 1 (ORF1) stop codon.

Several viral mRNAs contain elements that promote non-canonical modes of initiation, independent of eIF2 and/or eIF4F, to evade the influence of innate immune effectors that limit canonical cap-dependent initiation. These elements include IRESs in the 5′-untranslated region (5′ UTR) of positive-sense RNA genomes and in the intercistronic regions of dicistronic RNA genomes (discussed in more detail below) and upstream ORFs that regulate the initiation of translation of downstream ORFs [16].

3′-Cap Independent Translation Enhancer (3′-CITE) elements occur in the 3′-terminal regions of the genomes of members of several genera of *Tombusviridae* [17]. They are structurally diverse and function by distinct mechanisms that include binding directly to subunits of eIF4F or to the 80S ribosome or its 60S subunit, and then engaging with sequences proximal to the 5′-terminus via long-range base pairing, thereby directing the translation apparatus to the 5′-end of the mRNA in a cap-independent manner. Many of the viral translational control elements discussed by Khan and Fox [13] are not known to have cellular homologues, but repressive elements related to the Gamma-interferon-Activated Inhibitor of Translation (GAIT) element that occurs in several inflammation-related human mRNAs have been recently identified in respiratory syncytial virus and two coronaviruses. Members of the subgenus *Sarbecovirus* of genus *Betacoronavirus* also contain a GAIT-related Sarbecoviral Pan-End Activating RNA (SPEAR) element that is required for the inducible translation of coronavirus subgenomic mRNAs rather than for suppressing translation.

## 5. IRES Structure and the Mechanism of IRES-Mediated Initiation of Translation

Viral IRESs, which are structurally and mechanistically diverse, have been assigned to six major groups [18]. This classification excludes several less-well-characterized groups of IRESs, e.g., those in retrovirus, iflavirus, and some pegivirus mRNAs; moreover, additional IRES classes likely remain to be discovered because several validated IRESs have sequences and structures that are unrelated to those of known classes. The single-stranded positive-sense RNA genomes of dicistroviruses contain two principal open reading frames, ORF1 and ORF2, both translated following the initiation mediated by an IRES. The intergenic region (IGR) IRES promotes the translation of ORF2 by completely bypassing the conventional initiation process, enabling translation to proceed even when eIF2 and eIF4F are inactivated. Some dicistrovirus genomes diverge from this paradigm. For example, the Bemisia-associated dicistrovirus 2 (BaDV-2) IGR IRES has an atypical structure, and the upstream ORF1 is split into the overlapping ORF1a and ORF1b, so that the translation of the complete non-structural polyprotein would require a −1 frameshift. Chen et al. [19] confirmed that such a frameshift does occur and identified sequence determinants of this process. They also determined that the IGR IRES is only 140 nt long and differs from conventional IGR IRESs that are 190–200 nt long in that it does not bind in a direct, factor-independent manner to ribosomes or to ribosomal subunits, initiates translation at an AUG codon, and requires at least a subset of initiation factors. These findings emphasize both the diversity of mechanisms used by IGR IRESs and the diversity of IRES structures.

Type 1 IRESs occur in the genomes of enteroviruses such as poliovirus and enterovirus A71. They are over 450 nt long and are located downstream of a 5′-terminal cloverleaf that is a *cis*-acting replication element required for negative-strand synthesis. Infection by enteroviruses induces a profound shut-off of cellular translation caused by the cleavage of eIF4G by a viral protease into an N-terminal fragment that binds eIF4E and PABP and a C-terminal fragment that binds eIF4A and eIF3, and by the dephosphorylation and consequent activation of the translation repressor 4E-BP. Type 1 IRESs bind directly to the C-terminal cleavage product of eIF4G, which is thought to recruit other factors (e.g., eIF4A) and the ribosomal 43S preinitiation complex. Ribosomal entry may be followed by initiation at the 3′-border of the IRES (yielding an accessory protein) or by scanning downstream to the initiation codon for the viral polyprotein. A distinctive characteristic of type 1 IRESs is their dependence on IRES *trans*-acting factors (ITAFs), which are cellular RNA-binding proteins that activate IRES function, likely by inducing conformational changes in the IRES, but that are not involved in cellular translation. Differences in the level of their expression may be sufficient to restrict viral translation in some cell types and may therefore influence tissue specificity and host range. Abedeera et al. [20] surveyed cellular proteins that bind to these IRESs and influence their function, including a panel of ‘negative ITAFs’ that bind to these IRESs and inhibit their activity, likely by impairing their functional interactions with positive ITAFs or with canonical components of the translation apparatus. Significantly, ITAFs are commonly nuclear proteins that are redistributed to the cytoplasm as a consequence of infection, which is promoted by the cleavage of components of the nuclear pore complex by viral proteases. Therefore, viral infection shapes the intracellular environment to favor viral translation.

Type 2 IRESs are ~450 nt long and were initially identified in picornaviruses of the genera *Cardiovirus* (e.g., encephalomyocarditis virus) and *Aphthovirus* (e.g., foot and mouth disease virus (FMDV)). Type 2 IRESs were recently also identified in the 5′UTRs of some caliciviruses, having likely been acquired from picornaviruses by recombination. Arhab et al. [21] characterized the mechanism of initiation on representative type 2 IRESs from grey teal calicivirus (GTCV) and *Caliciviridae* sp. isolate yc-13 (also known as red-crowned crane calicivirus (RaCV1)) by the in vitro reconstitution of the initiation process. The canonical components of the translation apparatus required for initiation on these IRESs are analogous to those required for initiation on picornavirus type 2 IRESs, but their ITAF requirements showed interesting differences: the activity of both IRESs was enhanced by Ebp1 (also known as ITAF_45_, and initially identified as an FMDV-specific ITAF), whereas the GTCV IRES was further stimulated by the pyrimidine tract-binding protein (PTB), and the initiation mediated by the RaCV1 IRES was enhanced by Ebp1 and poly(rC)-binding protein 2 (PCBP2), previously thought to be specific to type 1 IRESs. These observations concerning overlapping ITAF requirements suggest that the mechanisms of initiation on type 1 and type 2 IRESs may be even more closely related than previously assumed. The analysis of calicivirus genomes revealed that many of those that contain type 2 IRESs also contained a 197–317-codon-long open reading frame (ORF1*) that overlapped with ORF1, the principal open reading frame. The use of overlapping reading frames is therefore another strategy employed by caliciviruses to maximize the exploitation of the coding capacity of their genomes, in addition to the synthesis of a polyprotein that is proteolytically processed to yield mature non-structural proteins, the synthesis of subgenomic mRNAs encoding capsid proteins, and termination–reinitiation to enable the synthesis of a minor capsid protein.

## 6. eIF4E-Independent Initiation Mediated by an Alternative Cap-Binding Complex

The primary human immunodeficiency virus 1 (HIV-1) transcript is capped, polyadenylated, and extensively spliced to yield over fifty mRNAs that encode fifteen proteins [22]. Infection leads to the transient activation of PKR followed by the repression of its activity, and the inhibition of the canonical initiation process as a result of HIV protease-mediated cleavage of eIF4G1, eIF3d and PABP, and the promotion of 4E-BP dephosphorylation by the HIV-1 Vpr protein, leading to the sequestration of eIF4E. Boris-Lawrie et al. [23] reviewed the alternate eIF4E-independent mechanisms of translation initiation employed by HIV-1, including IRES-mediated initiation, but they focused on the role of an alternative cap-binding complex that binds preferentially to a hypermethylated cap structure. The guanosine of the cap structure of unspliced HIV-1 transcripts is hypermethylated to trimethylguanosine (TMG) in a reaction that is promoted by RNA helicase A (also known as DHX9), and leads to the stable binding of an alternative cap-binding complex that consists of CBP80 and NCBP3 subunits. This complex recruits the 43S complex possibly via interactions with eIF3, but many details of this mechanism remain to be elucidated.

## 7. Conclusions

Many of the mechanisms by which host cells restrict the translation of viral mRNAs and by which viruses evade these restrictions and subvert the translation apparatus have been identified, but several of them remain incompletely characterized. The papers in this Special Issue contribute new insights into and review progress in understanding these processes. The various reports describe the mechanisms of action of different restriction factors, the mechanisms of viral translation, the role of viral proteins in counteracting the restriction of viral translation by cellular factors, and the role of viral RNA sequences and structures in evading innate immune antiviral responses and in enabling viral mRNAs to usurp the translation apparatus. These papers also highlight unresolved questions, emphasizing that much remains to be learnt on this topic and that further relevant insights can be anticipated.
